# *Konjac* flour-mediated gut microbiota alleviates insulin resistance and improves placental angiogenesis of obese sows

**DOI:** 10.1186/s13568-023-01646-4

**Published:** 2023-12-12

**Authors:** Deyuan Wu, Wenyu Xiong, Shuo Ma, Jinxi Luo, Hongxuan Ye, Shuangbo Huang, Fuyong Li, Xi’en Xiang, Qiling Chen, Binghui Gao, Jinping Deng, Yulong Yin, Chengquan Tan

**Affiliations:** 1 Guangdong Provincial Key Laboratory of Animal Nutrition Control, National Engineering Research Center for Breeding Swine Industry, Institute of Subtropical Animal Nutrition and Feed, College of Animal Science, South China Agricultural University, Guangzhou, 510642 Guangdong China; 2 Joinsha Animal Health Products (XIAMEN) CO., LTD. Xiamen, Fujian, 361000 China; 3 National Engineering Laboratory for Pollution Control and Waste Utilization in Livestock and Poultry Production, Institute of Subtropical Agriculture, Chinese Academy of Sciences, Changsha, 410125 Hunan China

**Keywords:** *Konjac* flour, Fecal microbiota transplantation, SCFA, Insulin resistance, Placental angiogenesis

## Abstract

Our previous study revealed that dietary *konjac* flour (KF) could remodel gut microbiota and improve reproductive performance of sows, but its underlying mechanisms remain unclear. This experiment aimed to investigate how dietary KF improves reproductive performance of obese sows. Here, 60 sows were assigned into three groups according to their backfat thickness: normal backfat sows fed with control diet (CON-N), high backfat sows fed with control diet (CON-H) and high backfat sows fed with KF inclusion diet (KF-H). The characteristics of sows and piglets were recorded. Next, fecal microbiota transplantation (FMT) was performed on female mice, followed by recording the characteristics of female mice. The results showed that compared with CON-H group, KF-H group showed downtrend in stillbirth rate (*P* = 0.07), an increase in placental efficiency (*P* < 0.01) and average piglet weight (*P* < 0.01); coupled with a decrease in the values of homeostasis model assessment-insulin resistance (*P* < 0.01); as well as an increase in placental vascular density and protein expression of angiogenesis markers (*P* < 0.01). As expected, sows fed KF diets had improved abundance and diversity of gut microbiota. More importantly, compared with CON-H(FMT) group, KF-H(FMT) group showed improvement in reproductive performance and insulin sensitivity (*P* < 0.05), as well as an increase in placental labyrinth zone and protein expression of angiogenesis markers (*P* < 0.05). Furthermore, we found a content increase (*P* < 0.05) of SCFAs in both KF-H group sow and KF-H (FMT) group mice. Overall, KF supplementation could alleviate insulin resistance, promote placental angiogenesis, and ultimately improve the reproductive performance of sows via gut microbiota remodeling.

## Introduction

In order to provide the fetus with sufficient nutrition for growth and development (Valencia-Ortega et al. 2021), the maternal sensitivity to insulin will decrease with the progress of pregnancy (Sonagra et al. 2014). However, the natural progression of this metabolic mechanism can be disrupted by maternal obesity, thus exacerbating maternal insulin resistance and leading to adverse maternal and fetal outcomes (Getahun et al. 2010; Grieger et al. 2018). Excessive backfat thickness at day 109 of gestation has been reported to increase insulin resistance in perinatal sows, and decrease the number of live piglets and litter weight (Cheng et al. 2019). However, the mechanism of insulin resistance responses in perinatal obese sows to reproductive performance has not fully elucidated.

Prior to birth, all the nutrients and oxygen required for fetal growth come from the maternal, and the placenta is the unique medium for the fetus to absorb nutrients and release metabolic wastes from the mother (Macpherson et al. 2017; Wu et al. 2022), revealing that placental vascular development is an important factor in determining fetal growth (Huang et al. 2021b). Mesenchymal stromal cells isolated from gestationally diabetic human placentae showed insulin resistance and decreased angiogenesis (Mathew and Bhonde 2017), but whether there are similar results in sows is inconclusive.

Accumulated evidence suggests that abundant fiber consumption can reduce the risk of insulin resistance and/or increase insulin sensitivity (Breneman and Tucker 2013; Deng et al. 2021; Kwon et al. 2018). Our previous study also found that sow dietary supplementation with highly fermentable fiber *konjac* flour (KF) could cause changes of gut microbiota abundance and improve insulin resistance (Tan et al. 2016a). However, whether KF can improve the reproductive performance and insulin resistance of sows through gut microbiota and its metabolites has not been confirmed. As an effective means to reestablish gut microbiota, fecal microbiota transplantation (FMT) is regarded as a breakthrough in medical progress in recent years. Therefore, this study aimed to use the FMT technology to verify whether KF can alleviate insulin resistance of sows by regulating gut microbiota to promote placental angiogenesis and reproductive performance.

## Materials and methods

### Animals and experimental design

This study was conducted in Jiangxi WanNianXinXing Agri-animal Co., Ltd., China (Jiangxi, China). A total of 60 *Duroc* × *landrace* × *yorkshire* sows with a similar farrowing time were blocked by backfat at day 65 of gestation, and assigned to one of three treatment groups (*n* = 20 per treatment): normal backfat sows fed with control diet (CON-N) (Average backfat thickness = 17.9 mm), high backfat sows fed with control diet (CON-H) (Average backfat thickness = 22.1 mm) and high backfat sows fed with KF inclusion diet (KF-H) (Average backfat thickness = 21.4 mm), respectively. All diets are designed to meet or exceed the nutritional requirements of the sow as recommended by the National Research Council (NRC 2012). The composition and nutrient composition of the experimental diets are shown in Table 1.

**Table 1:** Ingredients and nutrient composition of experimental diets (as-fed basis)

Item	CON	KF
Ingredient, %		
Corn	34.78	32.78
Rice bran meal	10.00	10.00
Broken rice	16.67	16.67
Soybean meal (43% CP)	14.50	14.50
Triticale	20.00	20.00
*Konjac* flour (KF)	–	2.00
Limestone	1.10	1.10
Dicalcium phosphate	0.96	0.96
Sodium chloride	0.40	0.40
Lysine sulfate (70%)	0.10	0.10
Choline chloride	0.13	0.13
Threonine	0.05	0.05
Mildewcide^a^	0.05	0.05
Antiseptic^b^	0.08	0.08
Premix^c^	1.18	1.18
Calculated composition^d^, %		
NE, Mcal/kg	2.39	2.33
Crude protein	13.87	13.87
Ether extract	2.32	2.25
Crude fiber	3.22	3.59
Neutral detergent fiber	11.23	12.39
Acid detergent fiber	3.96	4.39
Calcium	0.72	0.72
Total phosphorus	0.63	0.62
Lysine	0.76	0.76
Methionine	0.24	0.24
Threonine	0.56	0.56
Tryptophan	0.16	0.16
Analyzed composition, %		
Crude protein	14.43	14.25
Crude fiber	2.98	3.48
Neutral detergent fiber	12.47	13.77
Acid detergent fiber	4.55	4.83

^a^The mildewcide mainly consists of 99.5% potassium propionate

^b^The antiseptic mainly consists of 99% sodium diacetate

^c^Provided the following per kilogram of diet: 12,000 IU vitamin A; 200 mg vitamin C; 4800 IU vitamin D_3_; 205 mg vitamin E; 3.6 mg vitamin K; 3.6 mg vitamin B_1_; 12 mg vitamin B_2_; 7.2 mg vitamin B_6_; 0.048 mg vitamin B_12_; 8.6 mg folic acid; 48.0 mg nicotinic acid; 0.6 mg biotin; 30.0 mg pantothenic acid; 10.0 mg Cu; 130 mg Fe; 60 mg Zn; 45 mg Mn; 0.3 mg I; 0.1 mg Co. Premix is provided by Xinxing Agriculture and Animal Husbandry Co., LTD., Wannian County, Shangrao City (Jiangxi, China)

^d^Chemical concentrations were calculated using feed component values from (NRC 2012). Amino acid levels in diets are expressed as totals

The sows were housed in separate barns and all sows had free access to water. Feeding was done once a day (05:30) at 2.0 kg in late gestation.15 days before parturition, the feeding rate was gradually increased to 2.5 kg. 7 days before parturition, the sows are moved from the gestation room to the farrowing room and the ventilation system keeps the room at a suitable temperature.

### Sample collection and reproductive performance analysis

The number and weight of each live piglet and stillborn piglet were recorded at the time of sow delivery. Placental efficiency was calculated by dividing the birth weight of the piglets by the weight of the placenta. Following the previous method (Huang et al. 2021a), the piglets were delivered with the umbilical cord tied with fine cotton thread and labeled with a number before the umbilical cord was broken. After each placenta was expelled and weighed, small pieces of placental tissue (*n* = 6 per group) were taken and placed in liquid nitrogen for rapid freezing, and then a portion of fresh unextruded placental tissue was cut and immediately fixed in 4% paraformaldehyde. On the 100th day of gestation, blood was collected from sows of each test group via ear vein before feeding. Blood samples were centrifuged at 3000×*g* for 10 min at 4 °C to obtain supernatant and stored at − 20 °C for analysis (*n* = 6). On day 100 of gestation, six sows in each treatment group were selected and fresh feces were collected directly by massaging the rectum and then immediately stored at − 80 °C for further analysis.

### H&E staining

The placental tissues of sows and female mice embedded in paraffin were cut into 5 μm thick sections and then stained for H&E. A projection microscope (Olympus CX41, Japan) was used to locate the area of placental tissue on the slide. Subsequently, the area of placental vessels in sows and the area of placental labyrinth zone (LZ) in mice were quantified by Image J software (National Institutes of Health, Bethesda, MD) to determine the relative number of placental vessels per unit tissue area and the relative area of placental LZ.

### Intravenous glucose tolerance test

On day 100 gestation of sows or day 12.5 gestation of female mice, an intravenous glucose tolerance test (GTT) was performed as previously reported (Bowe et al. 2014). Briefly, fasting blood samples were collected after the experimental animals fasted overnight; blood samples were collected from the tail vein 15, 30, 60, 90 and 120 min after intravenous or intraperitoneal injection of 0.5 g glucose-kg body weight^−1^ of 50% glucose solution and blood glucose was measured immediately with an automated glucose analyzer (Sinocare Inc., Changsha, China). The collected fasting blood samples were centrifuged at 4 ℃ and 1500*g* for 10 min and then stored at − 20 ℃ after removing the supernatant for further analysis of glucose and insulin concentrations. For each GTT, the area under curve (AUC) of glucose was calculated by linear interpolation of glucose concentrations between the measurements, using the fasting glucose concentration as the baseline.

### Chemical analyses

Plasma glucose concentrations were measured using the Glucose Dehydrogenase Activity Colorimetric Assay Kit (Nanjing Jiancheng Bioengineering Institute, Nanjing, China) and plasma insulin levels were measured using the ultrasensitive porcine insulin ELISA kit (MEIMIAN, Jiangsu, China) according to the manufacturer’s instructions. Insulin resistance and sensitivity were evaluated through homeostasis model assessment (HOMA):

$$ {\text{Homeostasis model assessment{-}insulin resistance }}\left( {\text{HOMA{-}IR}} \right) = \left( {{\text{Fasting insulin }}\left( {{\text{FPI}}} \right) \left( {{\text{mIU}}/{\text{L}}} \right) \times {\text{Fasting glucose }}\left( {{\text{FPG}}} \right)  \left( {{\text{mmol}}/{\text{L}}} \right)} \right)/22.5. $$

### Antibiotics treatment (ABX) and FMT

Three-week-old C57BL/6 mice (Bestest, China) were purchased and fed for one week. In the fourth week, mice were treated with antibiotics according to the protocol of Liu et al. (2019). Briefly, a mixture of antibiotics (meilunbio, Dalian, China) metronidazole (1 g/L), ampicillin (1 g/L), neomycin sulfate (1 g/L), and vancomycin (0.5 g/L) was added to the drinking water. The amount of water drunk by the mice (~ 5 mL/day) was measured early each morning to confirm that each mouse had consumed enough antibiotics. Next, female mice were subjected to FMT according to the method of Chen et al. (2020). Fresh feces collected from three donors (sows) of the CON-N, CON-H or KF-H groups, respectively, were used as a single source for the corresponding CON-N (FMT), CON-H (FMT) and KF-H (FMT) mice. The ABX female mice were gavaged at 0.2 ml of their respective donor fecal supernatant per mice every other day for 4 weeks.

### 16S rRNA amplicon sequencing, data processing, and analysis

At G100, fresh feces of donor sow were obtained (*n* = 6), followed by 16S rRNA analysis. 16S rRNA amplicon sequencing, data processing, and analysis followed our previous report (Wang et al. 2018). Briefly, total DNA was extracted from fresh feces (~ 50 mg) using the DNA Stool Mini Kit (51504, QIAGEN). After determining the DNA quality, the region V3 and V4 of 16 S rRNA genes were amplified by the universal primer: F, 5′-GTGCCAGCMGCCGCGG-TAA-3′; R, 5′-GGACTACHVGGGTWTCTAAT-3′. Then, amplicons were extracted from a 2% agarose gel, purification with the GeneJET Gel Extraction Kit (Thermo Scientific), and quantification using a Quant-iT PicoGreen dsDNA assay kit (6163, Invitrogen; USA). Then, Sequencing libraries were generated using NEB Next® Ultra™ DNA Library Prep Kit for Illumina (NEB, USA) following manufacturer’s recommendations and index codes were added. The library quality was assessed on the Qubit@ 2.0 Fluorometer (Thermo Scientific) and Agilent Bioanalyzer 2100 system. At last, the library was sequenced on an Illumina HiSeq platform and 250 bp paired-end reads were generated.

Bioinformatic analysis as previously described (Wang et al. 2018). Briefly, using QIIME2, we merged, applied quality control and clustered the 16S rRNA gene reads into operational taxonomic units (OTUs). Taxonomic groups were based on the Greengenes Data-base V.13_8 using closed reference to perform referenced-based OTU clustering. Sequences with ≥ 97% similarity were assigned to the same OTUs. We pick a representative sequence for each OTU and use the RDP classifier (http://rdp.cme.msu.edu/) to annotate taxonomic information for each representative sequence (confidence threshold of 70%). The analysis of rarefaction curve, alpha-diversity (Shannon, chao1, observed species and PD whole tree), and beta-diversity (principal coordination analysis, PCoA) based on bray_curtis were performed using R package Vegan. One-way ANOVA followed by post hoc Tukey’s tests was performed to determine if the compositions of microbiota differed between groups unless otherwise noted. Linear discriminant analysis (LDA) effect size was used to elucidate the differences of bacterial taxa, with an LDA score threshold ≥ 3 as an important contributor to the model.

### Western blotting

The placental tissues were lysed in RIPA buffer (CWBIO) on ice for 10 min. Then, the lysate was centrifuged at 12,000*g* for 10 min. The protein concentration was determined by BCA method (Beyotime). Identical amounts of protein samples were separated by SDS/PAGE, namely vascular endothelial growth factor A (VEGF-A), platelet endothelial cell adhesion molecule-1 (CD31) and β-actin proteins. and then were transferred to polyvinylidene fluoride (PVDF) membranes After blocking with TBS/T buffer containing 5% milk, the membranes were incubated with the primary antibodies against VEGF-A (19003-1-AP, Proteintech, USA, 1:1000), CD31 (A0378, Abclonal, China, 1:1000) and β-actin (4970, CST, USA, 1:1000). After washing, PVDF membranes were incubated with appropriate HRP-conjugated anti-rabbit IgG secondary antibody (AS014, Abclonal, China, 1:5000). The density of bands was quantified using the ImageJ software (National Institutes of Health, Bethesda, MD) and then normalized to β-actin content.

### Fecal SCFAs analysis

Fecal SCFAs concentrations were measured as previously reported (Liu et al. 2021). Briefly, fecal samples were thawed on wet ice, followed by placing 0.5 g of each fecal sample in a centrifuge tube with 1 mL of distilled water, and homogenizing the mixture by vortexing for 1 min. After crushing in an ultrasonic bath for 30 min, the mixture was centrifuged at 13,000 rpm for 10 min to obtain the supernatant, followed by pouring all the supernatant into a new 2 mL centrifuge tube, adding 20 μL of 25% metaphosphoric acid and 0.25*g* anhydrous sodium sulfate, and homogenizing the mixture thoroughly by vortexing for 1 min. After adding 1mL methyl tert-butyl ether (operated in the fume hood), the mixture was thoroughly homogenized by vortexing for 5 min, followed by centrifugation at 13,000 rpm for 5 min and collecting the upper methyl tert-butyl ether extract (operated in fume hood). After filtration through a 0.22 μm microporous membrane, the filtrate was placed in a sample bottle with a lined tube for fecal SCFAs analysis.

### Statistical analyses

Except the rates of stillbirth and embryo resorption were tested by chi-square, the remaining data were statistically analyzed using the independent t-test in SPSS 24.0 (SPSS, Inc, Chicago). All data were tested for normality by using the Kolmogorov–Smirnov test while homogeneity of variances was determined by using the Levene's test. The results are represented as histograms from GraphPad Prism (GraphPad Software Inc, San Diego, CA), with each bar representing the mean ± SEM. 0.05 < *P* < 0.10 indicated a statistical trend of change, and statistical difference was expressed as *P < 0.05 and **P < 0.01, respectively.

## Results

### Effects of KF supplementation on insulin sensitivity and reproductive performance of high backfat sows

**Fig. 1:**
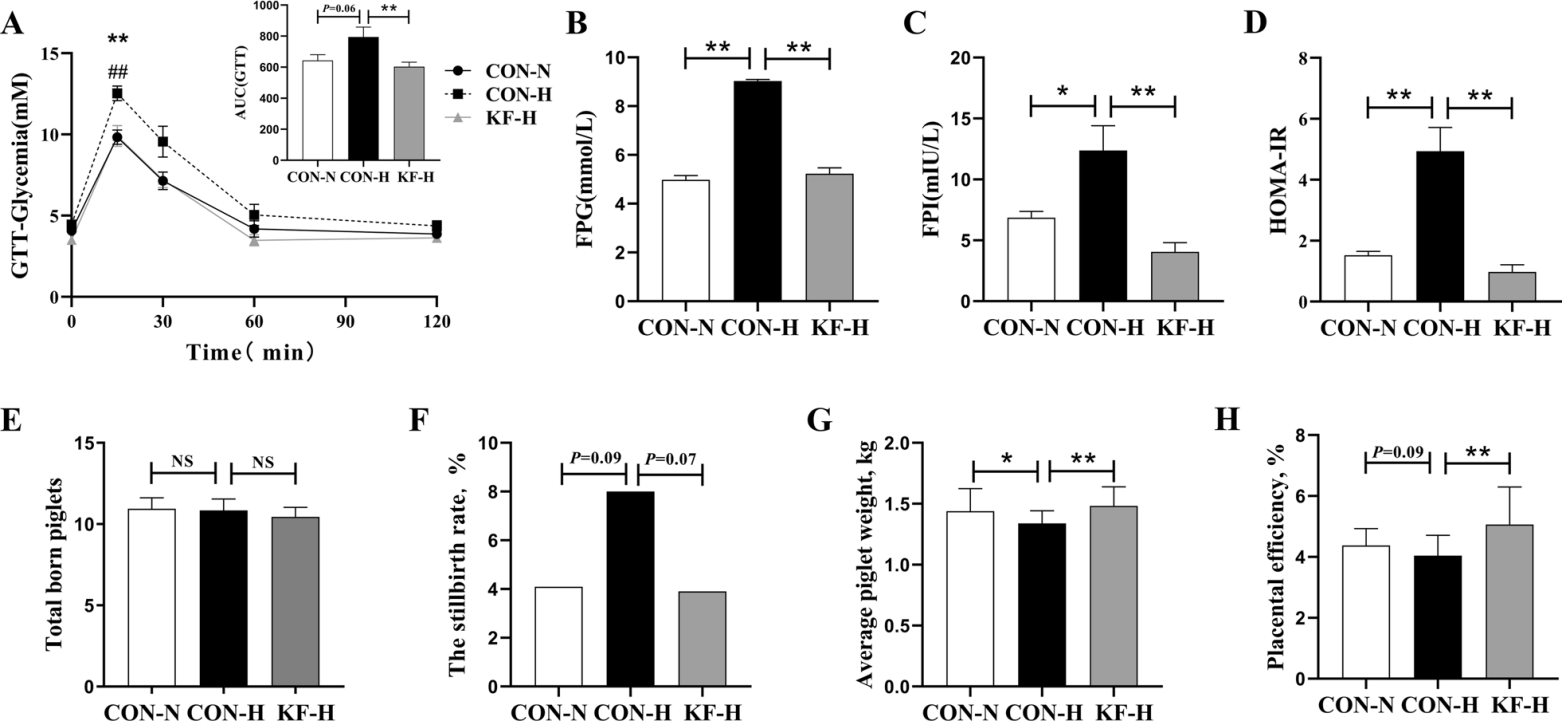
Effects of KF supplementation on insulin resistance and reproductive performance of sows. **A** Intravenous glucose tolerance test (GTT) from 0 to 120 min and area under the curve (AUC) of glucose from 0 to 120 min in GTT. (*n* = 6) **B** Fasting blood glucose level (FPG) (*n* = 6). **C** Fasting blood insulin level (FPI) (*n* = 6). **D** The values of homeostasis model assessment-insulin resistance (HOMA-IR) (*n* = 6). **E** Average piglet weight (*n* = 20). **F** Placental efficiency (*n* = 20). **G** Total born piglet (*n* = 20). **H** Stillbirth rate (*n* = 20). All data represent the mean ± SEM. Except that the stillbirth rate was tested by chi-square, the remaining data were statistically analyzed using the independent t-test. 0.05 < *P* < 0.10 indicated a statistical trend of change; *P < 0.05 and **P < 0.01

In Fig. 1A, the GTT results showed that at 15 min post glucose solution injection, CON-H sows were significantly (*P* < 0.01) higher than CON-N or KF-H sows in glucose level, and at 0–120 min, CON-H sow were significantly (*P* < 0.01) higher than KF-H sow and had an uptrend (*P* = 0.08) relative to CON-N sow in AUC of glucose. In Fig. 1B–D, insulin sensitivity indexes such as FPG, FPI and HOMA-IR were seen to be significantly higher in CON-H group than in CON-N or KF-H group (*P* < 0.05). Figure 1E–H showed the reproductive performance of sows in the three groups. In Fig. 1F–H, compared with CON-H group, CON-N group had a downtrend (*P* = 0.09) in stillbirth rate, the placental efficiency had a tendency to increase (*P* = 0.09), and a significant increase in average piglet weight (*P* < 0.05). Meanwhile, KF-H group was significantly higher CON-H group (*P* < 0.01) in average piglet weight and placental efficiency (Fig. 1G, H), coupled with a downtrend in the stillbirth rate of piglets (*P* = 0.07) (Fig. 1F).

### Effects of KF supplementation on gut microbiota of high backfat sows

**Fig. 2:**
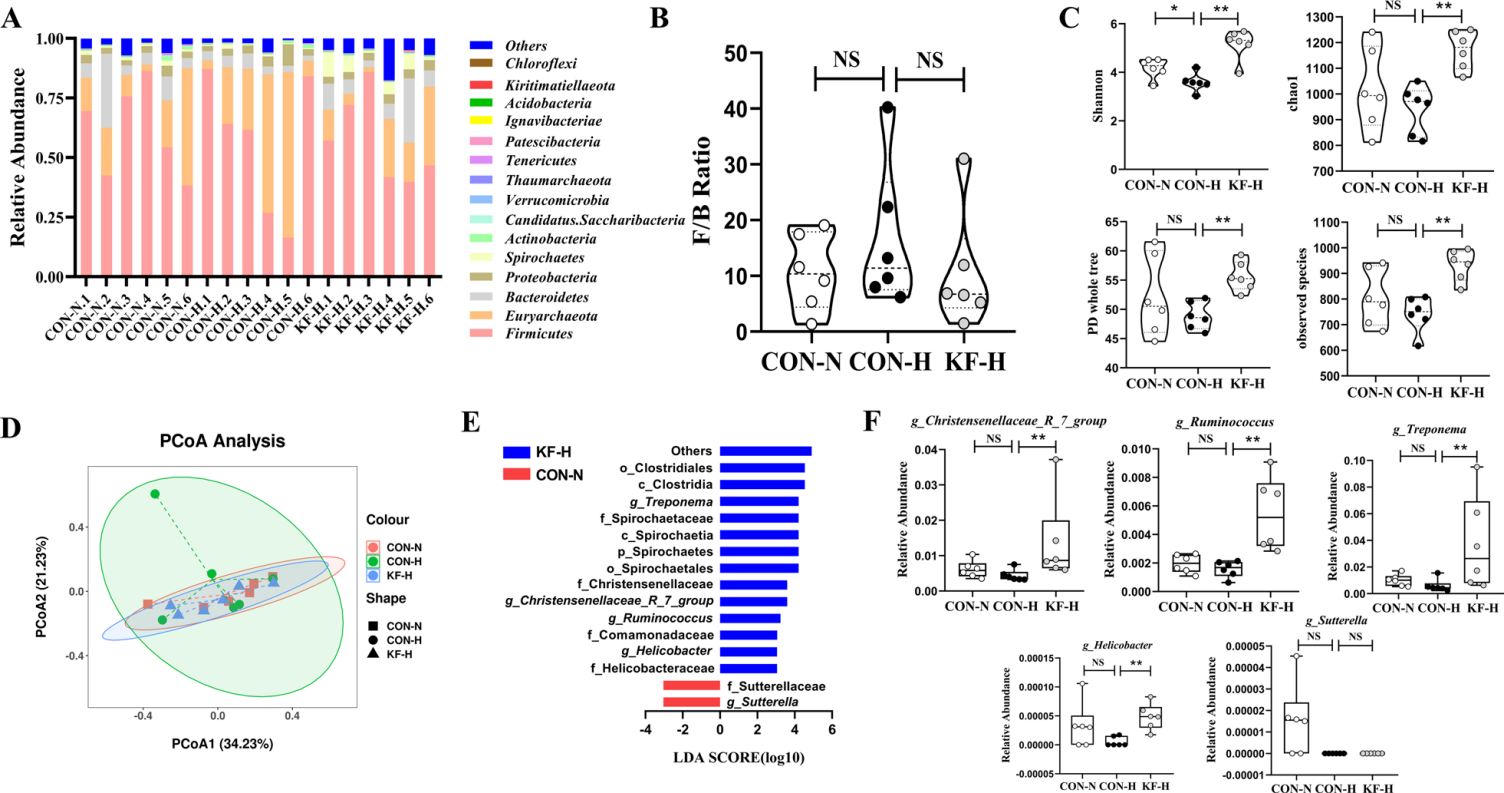
Gut microbial diversity and biomarkers. Each fecal sample was regarded as an experimental unit (*n* = 6 per group). **A** Abundance of gut bacteria at phylum level in different samples. **B** F/B Ratio of different groups. **C** α-diversity index (Shannon, chao1, PD whole tree, observed species). **D** PCoA of gut microbiota. **E** Linear discriminant analysis (LDA) effect size (LEfSe). **F** Relative abundance of core genera in different experimental groups. All data represent the mean ± SEM. Statistical differences in mean values were evaluated by independent t-test and by Mann Whitney test. 0.05 < *P* < 0.10 indicated a statistical trend of change; *P < 0.05 and **P < 0.01

Figure 2 presented the effect of dietary KF on gut microbial diversity and flora structure of sows. Figure 2A showed the structural composition of the gut microorganisms at the phylum level in different groups of sows. The five most abundant phyla in the fecal samples of the three groups were *Firmicutes*, *Euryarchaeota*, *Bacteroidetes*, *Spirochaetes* and *Proteobacteria*. We also counted the abundance ratios of the *Firmicutes* and the *Bacteroidetes*, respectively, and although F/B Ratio did not show significant differences from a statistical point of view, we could find that the F/B Ratio of the CON-H group was higher than that of the CON-N or KF-H groups from the results (Fig. 2B). As can be seen from Fig. 2C, the Shannon index of the CON-N group was significantly higher than that of the CON-H group (*P* < 0.01), while the Shannon, chao1, observed species and PD whole tree indices of the KF-H group were significantly higher than those of the CON-H group (*P* < 0.01). This highlights that diet KF reversed the reduction in α-diversity of gut microbiota in sows with high backfat. In Fig. 2B, the results of PCoA analysis based on bray_curtis distance showed that the samples in the CON-N and KF-H groups being obviously clustered, while the samples in the CON-H group differed more between groups. Figure 2E showed the biomarkers screened by the linear discriminant analysis effect size (LEfSe) analysis. We counted the relative abundance of the five core genera on this basis, and the results showed that the relative abundance of *Christensenellaceae_R_7_group*, *Ruminococcus*, *Treponema* and *Helicobacter* was significantly higher in fecal samples from the KF-H group than from the CON-H group (Fig. 2F) (*P* < 0.01).

### Effects of FMT supplementation on insulin resistance and reproductive performance in mice

**Fig. 3:**
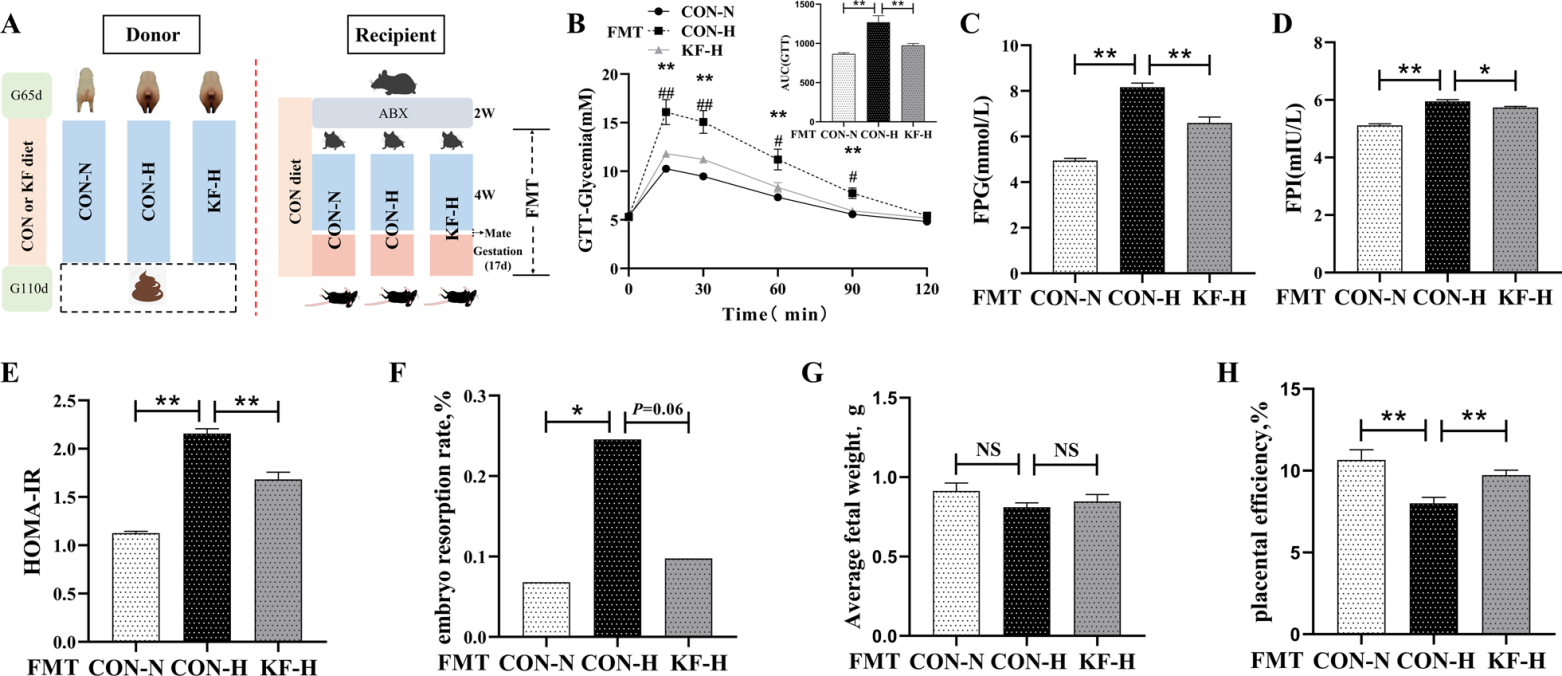
Insulin resistance and reproductive performance of female mice after fecal microbiota transplantation (FMT). Each sample of female mice was regarded as an experimental unit (*n* = 6 per group). **A** Diagram of FMT experiment model. **B** GTT from 0 to 120 min and AUC of glucose from 0 to 120 min in GTT. **C** FPG of female mice. **D** FPI of female mice. **E** The values of HOMA-IR of female mice. **F** Embryo resorption rate. **G** Average fetal weight. **H** Placental efficiency. All data represent the mean ± SEM. Except that embryo resorption rate was tested by chi-square, the remaining data were statistically analyzed using the independent t-test. 0.05 < *P* < 0.10 indicated a statistical trend of change; *P < 0.05 and **P < 0.01

As shown in Fig. 3B, after injection of glucose solution, the glucose level increased significantly in CON-H(FMT) female mice than in CON-N(FMT) or KF-H(FMT) female mice (*P* < 0.01). The AUC area of glucose at 0–120 min was also significantly higher in CON-H(FMT) group than in CON-N(FMT) or KF-H(FMT) group (*P* < 0.01). In Fig. 3C–E, CON-H(FMT) group was also seen to be significantly higher than CON-N(FMT) or KF-H(FMT) group (*P* < 0.01) in the insulin sensitivity indexes such as FPG, FPI and HOMA-IR. Figure 3F–H shows the reproductive performance of female mice in the three experimental groups. Compared with CON-H(FMT) group, CON-N(FMT) group exhibited a significant (*P* < 0.05) decrease in embryo resorption rate and a significant (*P* < 0.01) increase in placental efficiency. Meanwhile, KF-H(FMT) group also significantly increased (*P* < 0.01) in placental efficiency and had a downward trend (*P* = 0.06) in embryo resorption rate.

### Effects of KF supplementation on placenta morphology and angiogenesis in sows and mice

**Fig. 4:**
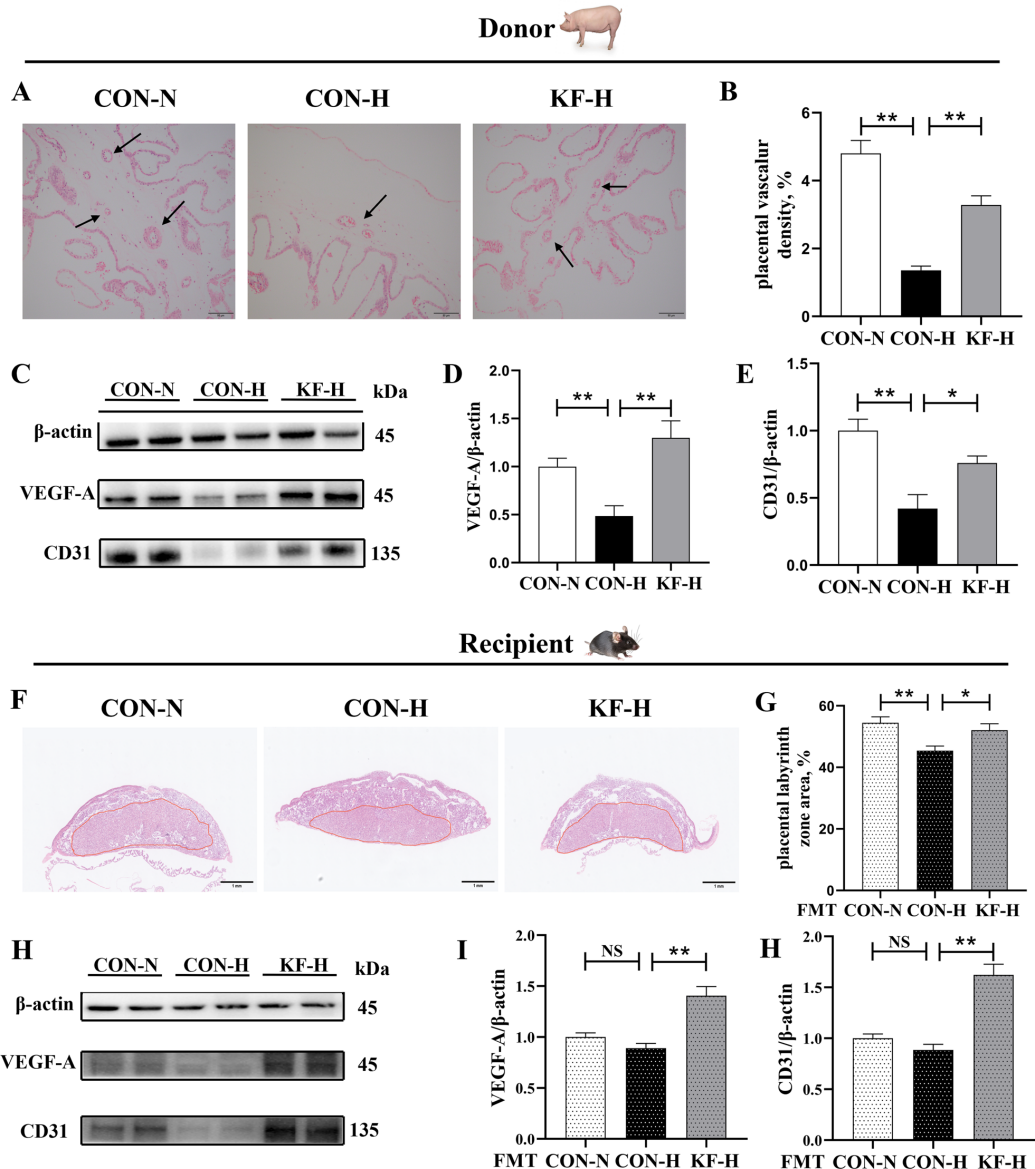
Placental angiogenesis of sows and female mice. Each placental tissue was regarded as an experimental unit (*n* = 6 per group). **A**, **B** H&E staining analysis of blood vessel density in the placental tissue of sows. The black arrow indicates the location of the placental blood vessels. **C**–**E** Western blotting analysis of protein expressions of VEGF-A and CD31 in the placentae of sows. **F**, **G** H&E staining analysis of the placental labyrinth zone of female mice. **H**–**J** Western blotting analysis of protein expressions of VEGF-A and CD31 in the placentae of female mice. All data represent the mean ± SEM. Statistical differences in mean values of all indexes were evaluated by using the independent t-test. 0.05 < *P* < 0.10 indicated a statistical trend of change; *P < 0.05 and **P < 0.01

In Fig. 4A, B, the placental vascular density of sows was shown to be significantly lower in CON-H group than in CON-N or KF-H group (*P* < 0.01). Protein expression analysis of angiogenesis markers in the placentae of sows found that CON-N and KF-H groups were significantly higher than CON-H group (*P* < 0.01) in the protein expression levels of VEGF-A and CD31 in (Fig. 4C–E). In addition, H&E staining of female mice placentae showed that compared with CON-H (FMT) group, the placental LZ area of female mice was significantly increased in both CON-N(FMT) group (*P* < 0.01) and KF-H(FMT) group (*P* < 0.05) (Fig. 4F, G). Protein expression analysis of angiogenesis markers in mice placentae showed that KF-H(FMT) group was significantly higher than CON-H(FMT) group (*P* < 0.01) in the protein expression levels of VEGF-A and CD31 (Fig. 4H–J).

### Effects of KF supplementation on fecal SCFA content

**Fig. 5:**
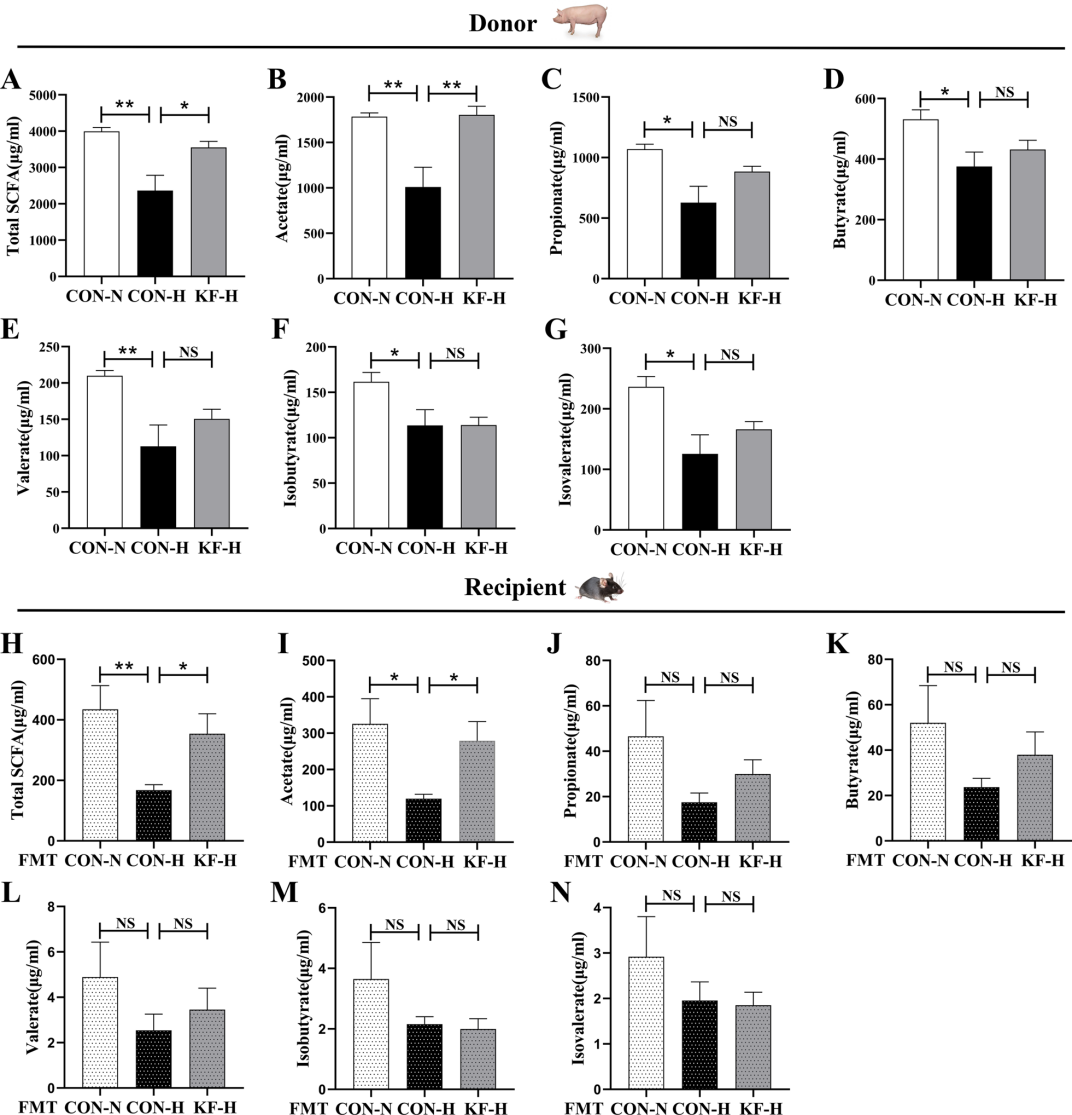
Fecal SCFA content in sows and female mice. Each fecal samples were regarded as an experimental unit (*n* = 6 per group). **A** Total SCFA content of sows. **B** Acetate content of sows. **C** Propionate content of sows. **D** Butyrate content of sows. **E** Valerate content of sows. **F** Isobutyrate content of sows. **G** Isovalerate content of sows. **H** Total SCFA content of female mice. **I** Acetate content of female mice. **J** Propionate content of female mice. **K** Butyrate content of female mice. **L** Valerate content of female mice. **M** Isobutyrate content of female mice. **N** Isovalerate content of female mice. All data represent the mean ± SEM. Statistical differences in mean values of all indexes were evaluated by using the independent t-test. 0.05 < *P* < 0.10 indicated a statistical trend of change; *P < 0.05 and **P < 0.01

In Fig. 5A–G, it was shown that compared with CON-H group, CON-N group had a significant increase (*P* < 0.05) in the contents of fecal total SCFA, acetate, propionate, butyrate, valerate, isobutyrate and isovalerate, as well as the SCFA in the KF-H group (*P* < 0.05). After FMT, fecal SCFA analysis also found that compared with CON-H(FMT) group, both CON-N(FMT) and KF-H(FMT) groups showed a significant (*P* < 0.05) increase in the contents of fecal total SCFA and acetate (Fig. 5H, I).

## Discussion

Increased insulin resistance is part of the physiological alterations of pregnancy, and increased maternal insulin resistance is thought to be caused by a combination of increased maternal obesity and the effects of placental hormone products (Salzer et al. 2015). Previous studies have demonstrated that thick backfat in sows at day 109 of gestation aggravates metabolic disorders of perinatal sows and decreases the number of live piglets and litter weight (Cheng et al. 2019). In addition, our study observed that high-fat-induced maternal obesity compromises placental angiogenesis and impairs neonatal glucose tolerance (Hu et al. 2019a, 2021b).

Dietary fiber has been widely reported to improve insulin resistance in obese maternal, and in a 3-day intervention in overweight and obese women, cereal fiber was observed to improve systemic insulin sensitivity (Weickert et al. 2006). Consistent with these studies, the present study found that insulin sensitivity and reproductive performance were significantly improved in high-backfat pregnant sows fed KF. In our previous studies, KF was identified as a highly fermentable dietary fiber, and as a refractory carbohydrate, KF can exert various health benefits based on its physicochemical properties, such as interacting with and influencing the gut microbial composition (Gill et al. 2021; Tan et al. 2016b; 2017). Our study also found that feeding KF improved the gut microbiota of pregnant sows, reversing the high backfat induced gut microbiota disturbances, including increased gut microbial diversity and promoting colonization of beneficial bacteria. F/B Ratio has been considered a marker of intestinal flora dysbiosis in some studies, and Jin et al. found significantly elevated F/B Ratio in the stool of pregnant women with preeclampsia (Jin et al. 2022). We also found in our experiment that F/B Ratio was also elevated in the CON-H group compared to the CON-N and KF-H groups. In addition, by comparing the abundances of the core genus, we found that dietary KF could improve the abundances of *Christensenellaceae_R_7_group* and *Ruminococcus*. *Christensenellaceae_R_7_group* has been proved to play an important role in intestinal immunity (Hu et al. 2019b; Wang et al. 2022). It is significantly negatively correlated with metabolic diseases such as body mass index and inflammation (Waters and Ley 2019). Goodrich et al. believed that increasing the abundance of *Christensenellaceae_R_7_group* in the intestine is beneficial to body health (Goodrich et al. 2014). *Ruminococcus* is a key bacterium for the digestion of resistant starch (Hong et al. 2022). Zhang et al. found that *Ruminococcus* can produce SCFA in the gut tract and reduce and improve gut inflammation (Zhang et al. 2017). Therefore, we hypothesize that dietary KF supplementation may alleviate insulin-resistant adverse pregnancy outcomes by improving the gut microbiome of sows and promoting SCFAs production.

FMT targeting gut microbiota is a new experimental approach in medical strategies (Bakker and Nieuwdorp 2017; de Groot et al. 2017). In recent landmark studies, patients with metabolic syndrome receiving FMT from healthy lean donors were shown to actually achieve metabolic improvement (de Groot et al. 2020; Kootte et al. 2017). In current study, fecal grafts from sows of different experimental groups were transplanted into female mice, and the FMT female mice showed similar metabolic status and performance indexes as the donors. The insulin sensitivity and reproductive performance were significantly improved in KF-H (FMT) mice compared with the mice receiving feces from CON-H sow. As an important organ linking maternal and fetus (Tan et al. 2022), placenta is accompanied by a large amount of angiogenesis during its formation, enabling the transfer of nutrition and energy from maternal to fetus (Hu et al. 2021a). Some studies have shown that insulin resistance can inhibit blood vessel dilatation, leading to cardiovascular diseases such as hypertension. It also plays a role in the damage and repair of blood vessels and the occurrence of atherosclerosis (Hill et al. 2021; Laakso and Kuusisto 2014; Lee et al. 2022). Our results showed a significant increase in placental blood vessel density of KF-H sows and in the placental LZ of KF-H(FMT) female mice. In female mice, placental LZ exchanges nutrients and gases between maternal and fetus. The placental zone has a complex structure, and defects in morphogenesis can compromise substrate exchange, thereby affecting fetal growth and viability (De Clercq et al. 2020; Kretschmer et al. 2020). In addition, we also found a significant increase in the expression levels of VEGF-A and CD31 in the placentae of KF-H sows and KF-H(FMT) mice. The similar results between sows and female mice further confirmed that KF may improve insulin resistance and adverse pregnancy in obese sows by mediating gut microbiota.

Moreover, we measured SCFAs in the feces from sows and female mice, and both KF-H sows and KF-H(FMT) female mice were found to have a significant increase in fecal total SCFA and acetate contents. As a product of gut bacterial metabolism, SCFA is particularly important in the diagnosis of gut dysbiosis (Ziętek et al. 2021). In addition, SCFAs have been reported to act as signaling molecules that regulate the body’s energy homeostasis through G-protein receptors and are therefore critical for the developing fetus, its future metabolic fate and maternal health. (He et al. 2020). Overall, our results indicated that dietary KF can alleviate insulin resistance of pregnant sows by increasing gut bacterial metabolites, thereby promoting placental vascular development and improving reproductive performance.

## Data Availability

The link of gut microbiome data is: accession PRJNA1005452.
